# Acute hydrops with a 180-degree massive edematous cavern demonstrated by three dimensional view of anterior segment optical coherence tomography in a patient with pellucid marginal corneal degeneration, a case report

**DOI:** 10.1186/s12886-018-0757-7

**Published:** 2018-04-13

**Authors:** Andrew Winegarner, Yoshinori Oie, Kohji Nishida

**Affiliations:** 1 Department of Ophthalmology, Osaka University Graduate School of Medicine, Room E7, Yamadaoka 2-2, Osaka, 565-0871 Japan; 2 Kansas University Medical Center, School of Medicine, Kansas City, KS USA

**Keywords:** Acute hydrops, Corneal perforation, Pellucid marginal corneal degeneration, Anterior segment optical coherence tomography, Gonioscopic view

## Abstract

**Background:**

Pellucid marginal corneal degeneration is a non-inflammatory disorder complicated by severe inferior corneal thinning. The central portion of the cornea, consequently, appears to protrude outwards, decreasing vision by means of an irregular stigmatism. Additionally, acute hydrops can occur in case of Descemet’s membrane rupture.

**Case presentation:**

A 41-year-old Japanese woman presenting with severe visual loss in the left eye was examined and observed to have had full thickness corneal perforation as well as a Descemet membrane rupture with massive edema in the corneal stroma. Anterior segment optical coherence tomography-based corneal topography revealed a distorted crab claw sign indicating pellucid marginal corneal degeneration. The Descemet membrane rupture allowed acute hydrops to occur which was especially noteworthy given the scale of edema present within the stroma, rotating 180 degrees along the limbus, causing a smiley-face like lesion. We visualized it via a gonioscopic three-dimensional optical coherence tomography to build a three-dimensional video. Patient history revealed a previous acute hydrops in the right eye as well, which was ultimately treated with anterior lamellar keratoplasty, suggesting the pellucid marginal corneal degeneration had a classic bilateral involvement, which was also characterized with bilateral acute hydrops.

**Conclusion:**

This appears to be a very rare and interesting presentation of bilateral pellucid marginal degeneration, wherein not only acute hydrops formed bilaterally, but the cavity within the cornea stroma was exceptionally large with an unusual shape. Using the gonioscopic three-dimensional optical coherence tomography imaging, we were able to easily visualize the massive intrastromal cavern, and appropriately planned the crescent-shaped anterior lamellar keratoplasty. The 3d video constructed using this data is particularly elucidative compared to 2d images. As such, we recommend utilizing 3d imaging in cases where more conventional topography is not as explanatory with respect to precise nature of deformation.

**Electronic supplementary material:**

The online version of this article (10.1186/s12886-018-0757-7) contains supplementary material, which is available to authorized users.

## Background

Pellucid marginal corneal degeneration (PMCD) is a non-inflammatory bilateral, inferior, peripheral corneal thinning disorder. Anterior corneal protrusion occurs above a band of thinning located 1 to 2 mm from the limbus [1]. The irregular shape decreases vision and causes irregular astigmatism. It also has been reported that eyes with typical PMCD have a fast triangular pattern for trefoil, an inferior slow pattern for coma, a positive spherical aberration regarding higher order aberrations, and a two-tailed comet-like pattern with a typical inferonasal and inferotemporal orientation with Landolt ring simulation [2]. Corneal thinning can lead to acute hydrops, rupture of Descemet’s membrane, and subsequent infiltration of aqueous humor into the corneal stroma. Spontaneous corneal rupture also has been reported during acute hydrops in patients with keratoconus or PMCD [3].

We describe a PMCD patient with full thickness corneal perforation followed by acute hydrops with a massive edematous cavern, which we visualized with anterior-segment optical coherence tomography (AS-OCT) gonioscopic view. Using the images, we created a 3d video, which proved much more elucidative than 2d images, to help plan surgical treatment.

## Case presentation

**Fig. 1:**
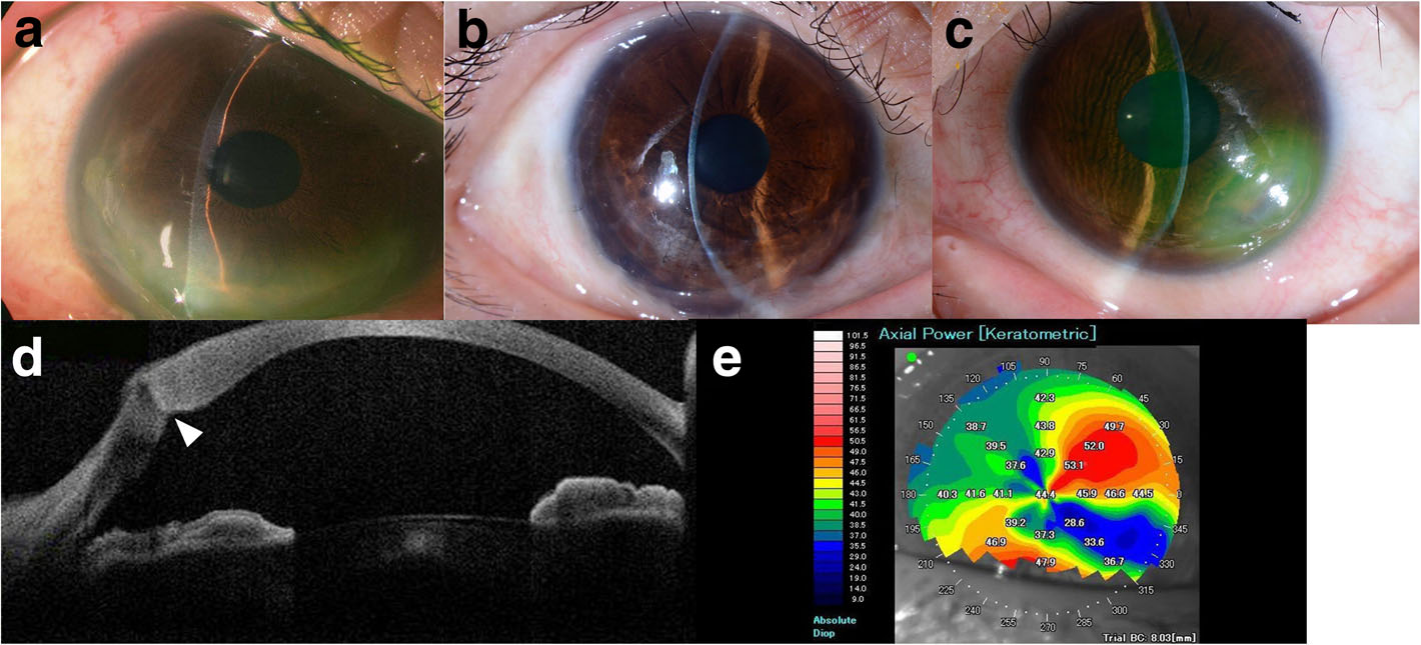
**a** A slit-lamp photograph taken in a previous hospital showed an extremely shallow anterior chamber and a corneal perforation resulting from acute hydrops in the right eye. **b** A slit-lamp photograph of the right eye obtained at the first visit showed a corneal scar in the inferior cornea. **c** A slit-lamp photograph of the left eye obtained at the first visit showed the corneal perforation and edema inferotemporally. **d** AS-OCT clearly showed a rupture in Descemet’s membrane. **e** AS-OCT topography showed a distorted crab-claw sign, indicating PMCD

A 41-year-old woman was referred for ocular pain, corneal opacity, and visual disturbance in her left eye. The corrected distance visual acuity (CDVA) in the left eye decreased to 20/1000, and the CDVA in the right eye was 20/25. She had a previous history of corneal perforation in the right eye, related to acute hydrops, treated by anterior lamellar keratoplasty (ALK) (Fig. 1a), and a postoperative corneal scar was observed (Fig. 1b). Slit-lamp and AS-OCT (SS-1000 CASIA, Tomey, Nagoya, Japan) examination of the left eye showed full thickness corneal perforation as well as rupture of Descemet’s membrane that means acute hydrops (Fig. 1c, d). Corneal topography using AS-OCT showed a distorted crab-claw sign indicating PMCD [3] (Fig. 1e).

**Fig. 2:**
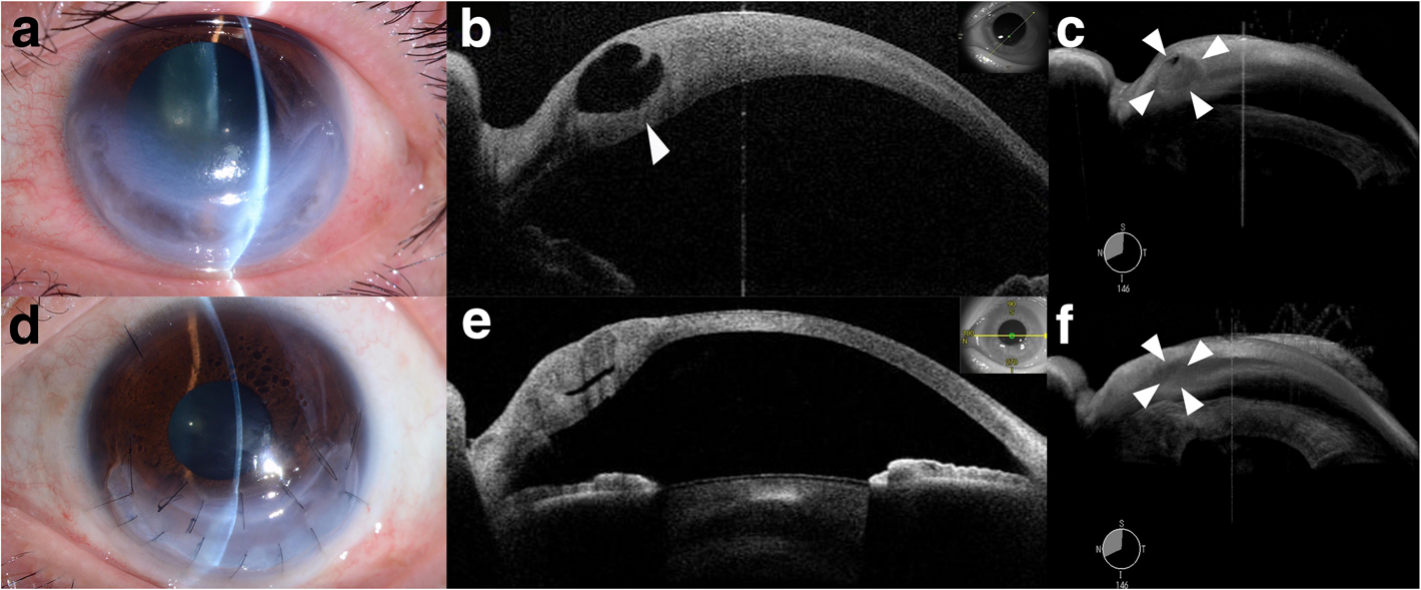
**a** A slit-lamp image obtained just preoperatively showed excessive edema that occupies as much as 180 degrees of the cornea. **b** Two-dimensional AS-OCT images showed a very large pathologic cavity in the left eye, with the arrow indicating the posterior corneal stroma prior to surgery. **c** a still from the 3d video demonstrating the edema in the preoperative condition. **d** A slit-lamp photograph obtained 1 month postoperatively showed decreased corneal edema. **e** Two-dimensional AS-OCT analyses showed the reduced abnormal corneal ventricle postoperatively. **f** A goniscopic view showed the postoperative resolution of the pathologic corneal structure compared to preoperatively

Conservative medical management was initiated, including therapeutic contact lens, occasional pressure patch, 0.3% ofloxacin ointment applied 4 times daily for 4 months. However, the acute hydrops did not resolve, rather the bleb grew. Slit-lamp examination showed that the corneal stromal edema had expanded into a massive cavernous crescent-shaped structure that extended 180 degrees along the limbus (Fig. 2a). AS-OCT images showed a large intrastromal cavity (Fig. 2b). A gonioscopic three-dimensional OCT view of the cornea and anterior chamber angle also clearly showed the large continuous corneal stromal cavity, whereupon the extent of the cavity could be properly appreciated (Fig. 2c, Additional file 1).

Our indication for surgery is acute hydrops resistant to conservative medical treatment. Therefore, an intracameral injection of 20% sulfur hexafluoride was administered to prevent further aqueous humor from entering the corneal stroma and facilitate resolution. However, instead of regressing, the edematous cavity continued to expand two weeks after injection. A crescent-shaped ALK was performed because the remaining posterior corneal stroma of the cavern was considered sufficiently thick to perform ALK under OCT observation (Fig. 2b, Additional file 1). After the anterior portion of the corneal stromal cavern was removed, a crescent-shaped corneal graft was sutured to the host cornea using 10-0 nylon during keratoplasty [4]. There were two difficulties of performing ALK. One difficulty was optimization of graft thickness because depth of stromal cavern was different between places in the patient. Thus, graft was gradually and carefully cut, and the thickness was successfully determined to adjust stromal cavern. The other difficulty was avoidance of anterior chamber collapse because of full thickness corneal perforation. Viscoelastic material was used to maintain anterior chamber during ALK. One month postoperatively, slit-lamp and OCT examinations showed that the corneal stromal ventricle structure had resolved markedly (Fig. 2d, e). The CDVA recovered to 20/29. A gonioscopic view clearly showed postoperative resolution of the pathologic corneal structure compared to preoperatively, conclusively demonstrating the surgical results (Fig. 2f, Additional file 1). Additionally, the 3d video allowed us to monitor the eye with greater precision in follow-ups.

## Discussion

We described bilateral corneal perforations resulting from acute hydrops, and one eye with a massive edematous cavernous structure observed by AS-OCT in a patient with PMCD. It has been reported that a corneal perforation can occur in cases with acute hydrops in patients with keratoconus or PMCD [3]. However, to the best of our knowledge, the current report is the first to describe full thickness corneal perforation accompanied by as large as 180-degree corneal stromal carven-like structure extending around half of the cornea, causing a sort of smiley-face shaped lesion. Moreover, the corneal perforations were so severe that bilateral ALKs were required in this case.

A previous report described spontaneous bleb formation due to a rupture in the stromal cleft, allowing a large edematous cavern to expand under the conjunctiva [5]. Where a break in Descemet’s membrane was close to the inferior limbus, and a corneal perforation resulted in aqueous humor to migrate into the subconjunctival space. The cornea in that case healed gradually as the result of treatment with a therapeutic soft contact lens and ointment in that report, however, in the current case, conservative therapies did not work due to case’s severity.

Most importantly, we showed the potential utility of AS-OCT. Topography showed the crab-claw pattern, which helped establish the diagnosis. Two-dimensional evaluation clearly showed the rupture in Descemet’s membrane and the corneal stromal cleft. However, the gonioscopic view facilitated a much more thorough understanding of the three-dimensional structure in the affected cornea, and helped us decide to perform ALK over a complete penetrating keratoplasty. Especially, the information was very helpful to adjust graft thickness to irregularly shaped host stromal carven. AS-OCT guided intrastromal fluid drainage with air tamponade was already reported [6]. However, three dimensional view demonstrated in this case is more informative than two dimensional evaluation.

## Conclusion

In conclusion, we described a case with a very severe corneal perforation by acute hydrops with a massive edematous cavernous structure that was refractory to medical treatment. AS-OCT 3D imaging was useful for examining the detailed structure of the pathologic lesion, for purposes of planning surgery, and for monitoring corneal status in follow-up examinations.

## Additional file

Additional file 1: 3d video of edema within the cornea. Video demonstrates in 3d the corneal edema with rotating image. (MP4 4741 kb)

## Abbreviations

ALK: Anterior lamellar keratoplasty
AS-OCT: Anterior Segment Optical Coherence Tomography
CDVA: Corrected distance visual acuity
PMCD: Pellucid marginal corneal degeneration
